# A Novel *IFITM5* Variant Associated with Phenotype of Osteoporosis with Calvarial Doughnut Lesions: A Case Report

**DOI:** 10.1007/s00223-021-00878-5

**Published:** 2021-06-22

**Authors:** R. E. Mäkitie, M. Pekkinen, N. Morisada, D. Kobayashi, Y. Yonezawa, G. Nishimura, S. Ikegawa, O. Mäkitie

**Affiliations:** 1 Folkhälsan Institute of Genetics, University of Helsinki, P.O. Box 63, FIN-00014 Helsinki, Finland; 2 Research Program for Clinical and Molecular Metabolism, Faculty of Medicine, University of Helsinki, Helsinki, Finland; 3 Department of Otorhinolaryngology Head and Neck Surgery, Helsinki University Hospital and University of Helsinki, Helsinki, Finland; 4 Department of Clinical Genetics, Hyogo Prefectural Kobe Children’s Hospital, Kobe, Hyogo Japan; 5 Department of Orthopaedic Surgery, Hyogo Prefectural Kobe Children’s Hospital, Kobe, Hyogo Japan; 6 Laboratory for Bone and Joint Diseases, RIKEN Center for Integrative Medical Sciences,, Yokohama, Japan; 7 Center for Intractable Disease, Saitama Medical University Hospital, Saitama, Japan; 8 Department of Molecular Medicine and Surgery and Center for Molecular Medicine, Karolinska Institutet, Stockholm, Sweden; 9 Children’s Hospital, University and Helsinki University Hospital, Helsinki, Finland

**Keywords:** IFITM5, SGMS2, Osteoporosis with cranial doughnut lesions, Cranial sclerosis, Osteomas, OI type V

## Abstract

Osteogenesis imperfecta (OI) and other decreased bone density disorders comprise a heterogeneous group of heritable diseases with skeletal fragility. Recently, it was discovered that mutations in *SGMS2*, encoding sphingomyelin synthetase 2, result in aberrant sphingomyelin metabolism and lead to a novel form of OI termed osteoporosis with calvarial doughnut lesions (OP-CDL) with moderate to severe skeletal fragility and variable cranial hyperostotic lesions. This study describes a Japanese family with the skeletal phenotype of OP-CDL. The affected individuals have moderately severe, childhood-onset skeletal fragility with multiple long-bone fractures, scoliosis and bone deformities. In addition, they exhibit multiple CDLs or calvarial bumps with central radiolucency and peripheral radiopacity. However, *SGMS2* sequencing was normal. Instead, whole-exome sequencing identified a novel *IFITM5* missense mutation c.143A>G (p.N48S) (classified as a VUS by ACMG). *IFITM5* encodes an osteoblast-restricted protein BRIL and a recurrent c.-14C>T mutation in its 5' UTR region results in OI type V, a distinctive subtype of OI associated with hyperplastic callus formation and ossification of the interosseous membranes. The patients described here have a phenotype clearly different from OI type V and with hyperostotic cranial lesions, feature previously unreported in association with *IFITM5.* Our findings expand the genetic spectrum of OP-CDL, indicate diverse phenotypic consequences of pathogenic *IFITM5* variants, and imply an important role for BRIL in cranial skeletogenesis.

**Supplementary Information:**

The online version contains supplementary material available at 10.1007/s00223-021-00878-5.

## Introduction

Osteogenesis imperfecta (OI) and other decreased bone density disorders comprise a phenotypically diverse group of heritable bone dysplasia with increased skeletal fragility, most commonly arising from genetic defects in type I collagen [1]. Rigorous research has, however, broken the narrative of collagen-related OI and unveiled several forms linked to defects in collagen-independent pathways that directly affect the extracellular matrix or bone cell function. In 2019, we expanded this spectrum by reporting novel genetic and phenotypic findings in a rare monogenic form of osteoporosis termed *osteoporosis with calvarial doughnut lesions* (OP-CDL, OMIM 126550) [2]. The disease is caused by mutations in the *SGMS2* gene, resulting in aberrant function of the encoded enzyme sphingomyelin synthetase 2 (SMS2) and sphingolipid metabolism, and consequently abnormal bone matrix mineralization and marked skeletal fragility. Depending on the type of the underlying mutation, affected individuals present with variably severe early-onset osteoporosis with spontaneous fractures and low bone mineral density (BMD) and typically without any of the classical extra-skeletal features of OI. In addition, they portray a distinctive feature of circular sclerotic cranial lesions, also referred to as calvarial doughnut lesions (CDLs). Although several families have been described so far—some prior to the genetic discovery—the disease is still extremely rare and its pathomechanisms incompletely understood [3–5].

Here, we describe a family with moderately severe skeletal fragility and multiple sclerotic skull lesions similar to the skeletal phenotype of OP-CDL. However, no pathogenic variant was found in *SGMS2*. Instead, whole-exome sequencing (WES) revealed a novel heterozygous mutation p.N48S in *IFITM5*, bringing new knowledge on the genetics behind OP-CDL and the phenotypic spectrum of *IFITM5* mutations.

## Patients and Methods

### Patients

We recruited a mother and her daughter with a monogenic skeletal dysplasia. Before participation in our study, both affected individuals as well as healthy family members signed a written informed consent. All clinical and genetic studies were performed according to ethically approved guidelines. Clinical data from medical records were collected retrospectively for all affected individuals in the family.

### Genetic Studies

Genomic DNA was extracted from peripheral blood leukocytes using standard methods or from saliva using Oragene DNA collection kit (DNA Genotek, Ottawa) according to the manufacturer's protocol. All five exons, 5′ UTR and 3′ UTR and a minimum of 30 bases of flanking introns in the *SGMS2* gene (NM_002335) were sequenced in two affected and two healthy family members by direct sequencing after polymerase chain reaction (PCR) amplification. Sequencing was done with an ABI 3730 DNA Analyzer (Applied Biosystems, Foster City, CA) and chromatograms analyzed using Sequencher v5.4.6 software.

**Fig. 1:**
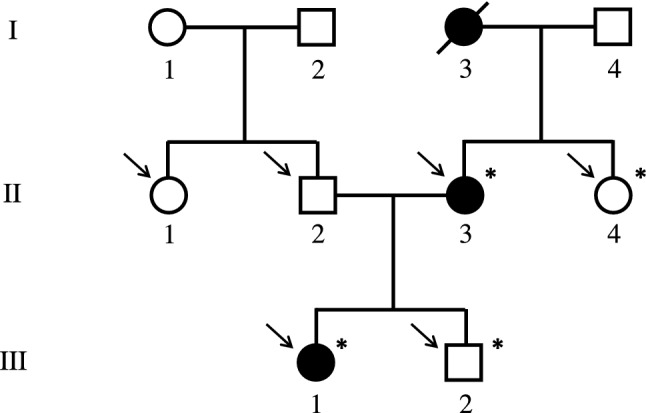
Pedigree of the Japanese family with a novel heterozygous *IFITM5* mutation p.N48S. Squares represent males, circles females, black mutation-positive, white mutation-negative, and slash a deceased individual. Asterisks indicate individuals included in WES analysis. Arrows indicate individuals Sanger sequenced for the *IFITM5* variant. Generations are shown with Roman numerals

For WES we included four family members from three generations: the index patient, her affected mother, her healthy father and her healthy brother (Fig. 1). WES was performed at Blueprint Genetics (Espoo, Finland) according to their standard methods (blueprintgenetics.com; Supplementary material). The procedure yielded × 178 median coverage of target bases. Reannotation was done with Annovar [6] and data analyzed with VarAFT 2.16 (http://varaft.eu).

Before exome-wide exploration for causative variants, we first evaluated all variants found in 23 known genes to underlie OI, bone fragility and primary osteoporosis (Figure S1) [7]. Second, we filtered variants in all genes and selected candidate variants with the following criteria: (1) heterozygous variants present in clinically affected subjects (II-3, III-1) and absent in clinically healthy subjects (II-2, III-2); (2) functional variants affecting coding regions or splice junction; and (3) an allele frequency < 0.1% in Genome Aggregation Database (gnomAD) database (v2 and v3) (www.gnomad.broadinstitute.org), the 1000 Genomes Project [8], The Single Nucleotide Polymorphism Database 153 (dsSNP153) (https://ftp.ncbi.nlm.nih.gov/snp/), the Sequencing Initiative Suomi (SISu) database (www.sisuproject.fi), The Human Genomic Variant Search Engine, Varsome [9] and Japanese multiomics panel (jMorp) (https://jmorp.megabank.tohoku.ac.jp/202008/). In silico predictions of the damaging capacity for missense variants were performed using SIFT (www.sift.jcvi.org), PROVEAN (http://provean.jcvi.org/index.php), PolyPhen2 (www.genetics.nwh.harvard.edu/pph2/), UMD Predictor (www.umd.predictor.eu), MutationTaster2 [10]), M-CAP (www.http://bejenaro.stanford.edu/mcap/), and CADD scores (www.cadd.gs.washington.edu). The variants' possible effects on protein conformation were evaluated using HOPE web server (http://www.cmbi.ru.nl/hope/). Segregation and trueness of the selected candidate variants were confirmed by Sanger sequencing (II-3, III-1, II-2, III-2, II-1, II-4) (Fig. 1). Primers and protocols are available upon request from the authors.

Copy number variation (CNV) was analyzed from WES data in all known disease genes by Blueprint Genetics (Espoo, Finland). These genes are supplemented with genes included in The Clinical Genomics Database (> 3350 genes, https://research.nhgri.nih.gov/CGD/) and the Developmental Disorders Genotype–Phenotype Database (DD2GP) (> 1640 genes, https://www.sanger.ac.uk/collaboration/deciphering-developmental-disorders-ddd/). The total number of genes considered as clinically associated in the CNV analysis is at least 3750.

## Results

### Case Report

The study involves a Japanese family clinically evaluated at Kobe Children’s Hospital, Japan, for an autosomal dominant fragile bone syndrome with CDLs (Fig. 1). The proband is a presently 18-year-old female with a history of multiple long-bone fractures starting at the age of two years. These were all result of low-energy trauma and included three separate left femur fractures, right femur, left tibia, and right tibia; no upper extremity fractures were recorded. The four femoral fractures were treated with recurrent corrective casts or intra-medullary osteosynthesis operations. No vertebral compression fractures were recorded. Her stature is otherwise normal with adult height of 165 cm (+ 1.2 SD). Her mother, presently 47 years, has a similar phenotype of early-onset skeletal fragility. She too has sustained multiple fractures, first at the age of six years. Despite the absence of vertebral compression fractures or exaggerated kyphosis, she has short stature with height of 133 cm (− 4 SD), partially perhaps accounted for by lower-limb deformities and spinal scoliosis. Neither patient exhibits other OI-related extra-skeletal characteristics (blue sclerae, dentinogenesis imperfecta, hearing impairment, or joint laxity), neurological symptoms related to basilar invagination commonly seen in OI, or any of the hallmark features of OI type V features (hyperplastic callus formation, calcification of forearm interosseous membrane, dislocation of the radial head or radiolucent metaphyseal bands) [8, 11]. Neither has received any bisphosphonate or other osteoporosis treatment at any point. The grandmother of the proband was reported to be similarly affected and had short stature but other detailed clinical data were unavailable.

**Fig. 2:**
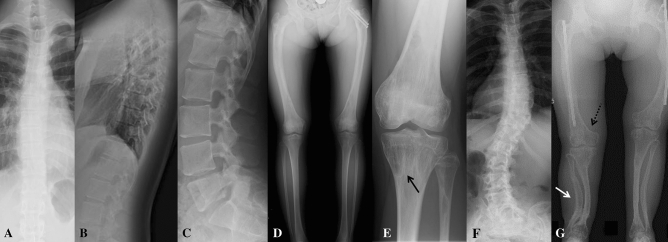
Skeletal radiographs of two patients with a novel heterozygous *IFITM5* mutation p.N48S. Images show (**A**) mild scoliosis, (**B**, **C**) normal thoracic and lumbar vertebral height without vertebral compression fractures, (**D**) mild femoral bowing with diaphyseal cortical thickening, and (**E**) metaphyseal osteopenia intermingled with sclerotic striations (arrow), cystic (tumor-like) lesions, and preserved cortical thickness of the distal femur and proximal tibia in an 18-year-old female; and (**F**) severe scoliosis and (**G**) long-bone deformity with metaphyseal widening (dashed arrow) and diaphyseal bowing (white arrow) in a 47-year-old female

**Fig. 3:**
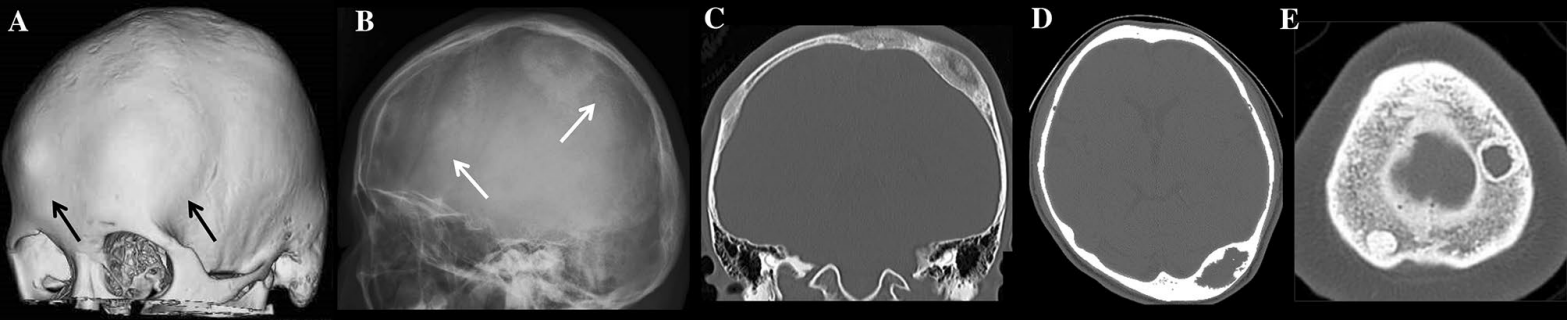
Cranial lesions in two female patients with a novel heterozygous *IFITM5* mutation p.N48S**.** (**A**) 3D CT showing osseous bumps of the right frontal and left parietal calvaria (black arrows); (**B**) skull radiograph showing doughnut lesions of the frontal and parietal regions (arrows), corresponding to ones in (**A**); and (**C**) Coronal CT showing parietal hyperostosis with central radiolucency in an 18-year-old female. (**D**) CT showing calvarial thickening with central radiolucency of the left occipital region in a 47-year-old female. (**E**) Cross-sectional CT image of an adult male patient with a heterozygous SGMS2 mutation p.R50* showing multiple sclerotic lesions

Skeletal radiographs show demineralized bones with moderate deformities (Fig. 2). The proband portrays mild bowing of the femora with diaphyseal cortical thickening, but no deformity of other long bones. Metaphyseal modeling of the tubular bones is normal. Bone mineralization is uneven with alternating areas of densely and poorly mineralized bone. The metaphyses are strikingly osteopenic, while the diaphyses are not evidently demineralized and have normal diaphyseal cortical thickness. It is notable that the metaphyseal radiolucencies of the knee are intermingled with sclerotic striations and cystic, tumor-like lesions. The spine shows only mild scoliosis and no vertebral compression fractures (Fig. 2B, C). In the mother, radiographs of the trunk and legs show more severe skeletal abnormalities with severe bowing of the femora, tibiae, and fibulae, and evident metaphyseal overmodeling of the tubular bones. The metaphyses are severely demineralized, while the bent diaphyses are associated with cortical thickening. Her spine shows mild scoliosis; no lateral spine images were available to assess vertebral height or shape. Lastly, both also exhibit several distinctive changes of the calvaria. Skull radiographs and cranial CT reveal multiple calvarial bumps with central radiolucency and peripheral radiopacity (Fig. 3).

**Fig. 4:**
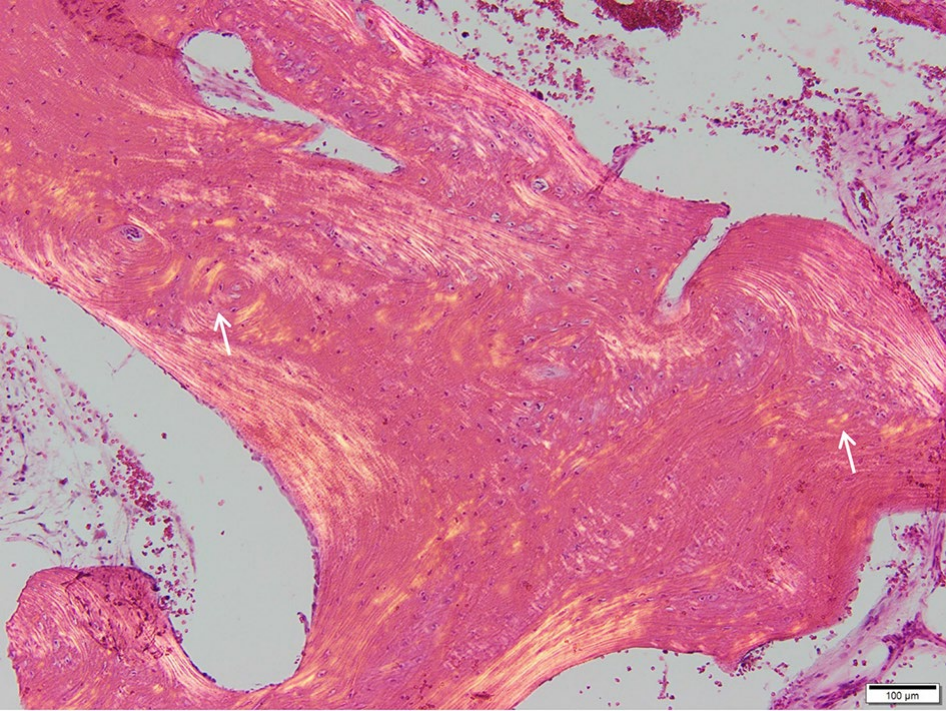
Bone biopsy sample from a female patient with a novel heterozygous IFITM5 mutation p.N48S. Images show an absence of the birefringent pattern of normal lamellar bone and the presence of fish-scale pattern (arrows) under polarized light

No bone mineral density (BMD) measures are available in either individual. Biochemistry of bone turnover markers, including alkaline phosphatase, have remained constantly normal. For the index, bone histomorphometry, performed on a bone biopsy taken during a hip joint surgery at age 10, portrayed an absence of the birefringent pattern of normal lamellar bone and the presence of fish-scale pattern under polarized light (Fig. 4).

### Genetic Findings

**Fig. 5:**
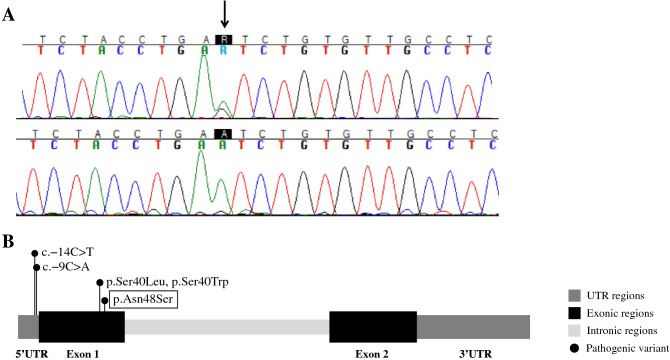
Genetic results in a family with a novel heterozygous *IFITM5* missense mutation p.N48S**.** (**A**) Sanger sequence image of the heterozygous point mutation in the index patient and a normal sequence in a healthy family member (II-4). (**B**) The four previously reported mutations in *IFITM5* linked to osteogenesis imperfecta. The novel mutation reported in this publication is marked by box

No pathogenic variants were found in *SGMS2* by Sanger sequencing. CNV analysis of known disease genes (> 3650), defined as single exon or larger deletions or duplications, did not reveal any potential variations probable to cause the disease. Screening of the WES data for pathogenic variants in the known OI and primary osteoporosis genes yielded a novel missense mutation c.143A > G (p.N48S) in exon 1 of *IFITM5* (Fig. 5). The variant is classified as a variant uncertain significance (VUS) according to the American College of Medical Genetics and Genomics (AMCG). Further filtering of the whole WES data for other single nucleotide variations (SNVs), small insertions and deletions (INDELs), yielded two candidate variants: one missense variant and one splice region variant. These two variants, however, were discarded for their close association with known human genetic diseases with different phenotypes to that of our family (Supplementary Table). Detailed filtering steps and the number of variants after each filtering step for a dominant disorder are shown in supplementary Fig. 1S.

The identified *IFITM5* missense variant (p.N48S) is novel with no carriers identified in the aforementioned databases. The variant is predicted to be damaging by all six prediction programs: damaging by SIFT (0.012), and PROVEAN (-4.9), M-CAP (0.310), probably damaging by PolyPhen2 (0.896), pathogenic by UMD Predictor (pathogenicity 90), and disease causing by MutationTaster2 (0.996). It also has a CADD score of 22.7. Automated analysis of the mutant protein by HOPE indicated that the residue is likely located in a transmembrane domain, is smaller than the wild-type residue, and this size difference can affect the protein's contacts with the lipid membrane. According to SwissProt, the 3D structure is different between WT, S40L, S40W, and N48S but unfortunately, the crystal protein structure of IFITM5/BRIL has not been resolved and not enough structural data is available to generate structural models of the protein. In addition, the mutant residue is more hydrophobic than the wild-type residue, which may affect its hydrophobic interactions with the membrane lipids. The mutation also lies in a 100% conserved residue and is likely damaging for the protein. The variant is also only eight amino acids from a previously published *IFITM5* locus known to underlie OI [11–13]. Lastly, in STRING analysis, we did not find direct connections between IFITM5 and SGMS2.

## Discussion

Osteoporosis with cranial lesions (OP-CDL) is a unique form of OI with moderate to severe skeletal fragility and multiple sclerotic cranial lesions. Recently, the genetic cause was identified as mutations in the sphingomyelin synthase 2–encoding *SGMS2* and consequent abnormal sphingomyelin metabolism [2]. Here we provide detailed description of two related individuals with the skeletal phenotype of OP-CDL but without a pathogenic variant in *SGMS2*. Instead, both harbor a novel heterozygous missense mutation p.N48S in *IFITM5*, a genetic defect previously primarily reported in OI type V and, in a few cases, OI type VI [1, 11–18]. These together expand the genetic background of OP-CDL, underline the importance of differential diagnostics and possibly insinuate a communication between collagen processing and sphingomyelin metabolism in cranial skeletogenesis.

BRIL is a bone-specific modulator of mineralization and mutations in its encoding gene *IFITM5* gene have previously been exclusively associated with OI type V or VI [11–18]. To date, only four different *IFITM5* mutations have been described [11–18] and the affected patients show great phenotypic variability depending on the contrasting effects of the different mutations. Patients with the recurrent c.-14C>T 5' UTR mutation, affecting the intracellular N terminus of the protein, represent the classical OI type V with interosseous membrane calcification, hyperplastic callus formation, radial head dislocation and characteristic metaphyseal bands [14, 15]. The exonic p.S40L and p.S40W mutations, affecting the coding region of the gene and the transmembrane domain of the protein, portray a phenotype resembling the recessive OI type VI with severe skeletal dysplasia and prenatal bowing of the long bones, broad metaphyses, and coxa vara, and histologically hyperosteoidosis [12, 13]. Recently, a fourth mutation c.-9C>A was described but at a young age of only one month, it is not possible to definitively ascertain the full of extent of the skeletal phenotype [16].

Our patients, though also harboring a mutation in the coding region in close proximity to the S40 mutations, have clinical presentations very different from these. Both exhibit skeletal fragility with postnatal onset of fractures, diaphyseal bowing, and normal metaphyseal modeling and, most distinctively, multiple CDLs. The patients lacked interosseous membrane calcification or hyperplastic callus formation but had relatively thick long-bone cortices [19]. Neither also has blue sclerae, cardiac symptoms, cystic vertebral lesions or other spinal anomalies—clinical features reported in patients with S40 mutations [12, 13]. This may, of course, be a characteristic of this single family and not represent the full clinical phenotype caused by this mutation. Further, prior reports have described delayed and uneven ossification of cranial bones in patients with *IFITM5* mutations, such as Wormian bones and granular lytic areas of craniofacial bones [18]. Dagdeviren et al. [20] also described a young female patient with a sclerotic granular bone pattern of the maxillary and mandibular bones with extensive dental problems and a mixture of osteosclerosis and osteolysis of the cranial base. However, she did not have CDL or overt calvarial hyperostosis.

The skeletal manifestation of the present family is classifiable into OP-CDL. However, there are phenotypic differences between the present family and *SGMS2*-related OP-CDL [2]. Bone fragility, bone deformities, and growth retardation are much more severe in *SGMS2*-related OP-CDL [2]. Also, vertebral compression fractures were a key finding, neurological complications are common, ALP levels tend to be elevated, and CDLs are more sclerotic. Furthermore, histological examination for bone show impaired mineralization or abnormal bone lamellarity.

Given the phenotypic similarities between *SGMS2*- and *IFITM5*-related OP-CDL it may be postulated whether the two syndromes share essential molecular connection or close relation between sphingomyelin metabolism and BRIL. One such hypothetic link may be SP7 (osterix), a transcription factor of osteoblast differentiation, whose expression is under the control of several signaling pathways, endoplasmic reticulum stress and epigenetic factors [21]. SP7 has an activator that acts cooperatively with specificity protein 1 (SP1) and zinc finger protein GLI2 to synergistically induce the BRIL promoter [22], and reports show that complete depletion of SMS2 in osteoblasts by Sp7 promoter-driven Cre-expressing mice (Sp7-Cre; SMS1-CKO; SMS2-KO) induces osteopenia through reduced bone formation [23]. Although our findings would support such interaction, and functional in vitro studies suggest BRIL to similarly partake in matrix mineralization, results from animal studies are unclear and partly conflicting [24–27]. Also, analysis for possible protein interactions with SwissProt did not find any connections between BRIL and SMS2. Therefore, these suggestions remain on a purely hypothetical basis and warrant further investigations.

We acknowledge certain limitation to our study, mainly concerning the lack of functional assessment of the molecular outcomes of the identified mutation. Also, we acknowledge that having clinical data from only one family provides a limited view on the full spectrum and severity of skeletal and possible extra-skeletal manifestations. Especially, more extensive bone histomorphometry data would have been very informative in determining detailed skeletal outcomes of the *IFITM5* mutation. Furthermore, future studies are recommended to address the role of *IFITM5* mutations in cranial pathology and its possible links to sphingomyelin metabolism. Lastly, we acknowledge the possibility of random segregation of this variant as only few family members were screened. With the number of tested individuals, the probability of such random segregation would be 1:8. However, taken together, we do consider our findings valuable and important given the novelty of the reported findings.

As conclusion, we report a novel *IFITM5* mutation presenting with OP-CDL with moderately severe skeletal fragility and deformities and notable cranial abnormalities, suggesting genetic heterogeneity in OP-CDL. *IFITM5*-related skeletal fragility should be considered in subjects with OP-CDL like skull lesions. Functional analyses evaluating possible connection between BRIL and sphingomyelin metabolism may shed light on the pathogenetic mechanisms.

## Supplementary Information

Below is the link to the electronic supplementary material.

Supplementary file1 (DOCX 99 kb)

## Data Availability

Primers and protocols are available upon request from the authors.
