# Supplementary material for: A Novel IFITM5 Variant Associated with Phenotype of Osteoporosis with Calvarial Doughnut Lesions: A Case Report
Source: Calcif Tissue Int. 2021 Jun 22;109(6):626–32. doi: 10.1007/s00223-021-00878-5 (PMC8531111; doi:10.1007/s00223-021-00878-5)
Supplement: Supplementary file 1 — Supplementary file1 (DOCX 99 kb) [file 223_2021_878_MOESM1_ESM.docx]

**A novel *IFITM5* variant associated with phenotype of osteoporosis with calvarial doughnut lesions: a case report**

Mäkitie RE^1,2^*, Pekkinen M^1,2^*, Morisada N^3^, Kobayashi D^4^, Yonezawa Y^5^, Nishimura G^6^, Ikegawa S^5^*, Mäkitie O^1,2,7,8^*****

***** REM and MP contributed equally to this work. # SI and OM contributed equally to this work.

^1^ Folkhälsan Institute of Genetics, Helsinki, Finland

^2^ Research Program for Clinical and Molecular Metabolism, Faculty of Medicine, University of Helsinki, Helsinki, Finland

^3^ Department of Clinical Genetics, Hyogo Prefectural Kobe Children’s Hospital, Kobe, Hyogo, Japan

^4^ Department of Orthopaedic Surgery, Hyogo Prefectural Kobe Children's Hospital, Kobe, Hyogo, Japan

^5^ Laboratory for Bone and Joint Diseases, RIKEN Center for Integrative Medical Sciences

^6^ Center for Intractable Disease, Saitama Medical University Hospital, Saitama, Japan

^7^ Department of Molecular Medicine and Surgery and Center for Molecular Medicine, Karolinska Institutet, Stockholm, Sweden

^8^ Children’s Hospital, University and Helsinki University Hospital, Helsinki, Finland

**Address all correspondence and requests for reprints to:**

Riikka E Mäkitie, MD PhD

Folkhälsan Institute of Genetics, P.O. Box 63,

FIN-00014 University of Helsinki, Helsinki, FINLAND

E-mail: riikka.makitie@helsinki.fi

Tel. +358-2941911

**Supplementary methods**

*Whole-exome sequencing*

Whole-exome sequencing was performed at Blueprint Genetics according to their protocol. Genomic DNA quality and quantity were assessed using electrophoretic methods. After assessment of DNA quality, qualiﬁed genomic DNA sample was randomly fragmented using non-contact, isothermal sonochemistry processing and puriﬁed with SPRI beads. DNA fragments were then end-repaired and sequencing adapters were ligated to both ends of the resulting fragments. Prepared DNA-Adapter libraries were size-selected with SPRI beads to ensure optimal template size and then ampliﬁed by ligation-mediated PCR (LM-PCR). The ampliﬁed sequencing library was puriﬁed using SPRI beads and a hybridization-capture method was applied for enrichment of whole exome and select non-coding regions (xGen Exome Research Panel with custom-designed capture probes, IDT). The enriched sequencing library was ampliﬁed by LM-PCR and puriﬁed using SPRI beads. The quality of the completed sequencing library was controlled by ensuring the correct template size and quantity and to eliminate the presence of leftover primer-dimers. Each captured library passing quality control was sequenced using the Illumina sequencing system with paired-end sequencing (150 by 150 bases). Sequencing-derived raw image ﬁles were processed using a base-calling software (Illumina) and the sequence data was transformed into FASTQ format.

Bioinformatics and quality control: The bioinformatics analysis began with quality control of raw sequence reads. Clean sequence reads of each sample were mapped to the human reference genome (GRCh37/hg19). Burrows-Wheeler Aligner (BWA-MEM) software was used for read alignment. Duplicate read marking, local realignment around indels, base quality score recalibration and variant calling were performed using GATK algorithms (Sentieon). The panel content was sliced from high-quality exome sequencing data acquired as presented above. The sequencing depth and coverage for the tested sample were calculated based on the alignments. The sequencing run included in-process reference sample(s) for quality control, which passed our thresholds for sensitivity and speciﬁcity. The patient's sample was subjected to thorough quality control measures as well, after which raw sequence reads were processed into variants by a proprietary bioinformatics pipeline. Copy number variations (CNVs), deﬁned as single exon or larger deletions or duplications (Del/Dups), were detected from the sequence analysis data using a proprietary bioinformatics pipeline, which processes aligned sequence reads. The diﬀerence between observed and expected sequencing depth at the targeted genomic regions was calculated and regions were divided into segments with variable DNA copy number. The expected sequencing depth was obtained by using other samples processed in the same sequence analysis as a guiding reference. The sequence data was adjusted to account for the eﬀects of varying guanine and cytosine content.

**Supplemental Figure 1. Workflow for analysis of whole-exome sequencing data from a Japanese family with an autosomal dominant form of osteoporosis with calvarial doughnut lesions.**

**Supplementary Table. Other potential heterozygous variants in a Japanese family with autosomal dominant osteoporosis with calvarial doughnut lesions.**

| **Gene** | **Variant (hg19)** | **Variant type** | **Transcript** | **MAF gnomAD^†^** | **CADD** | **SIFT** | **Provean** | **Polyphen 2** | **Mutation Taster2** | **LRT** | **M-Cap** | **UMD Prediction** | **Syndromes linked** |
| --- | --- | --- | --- | --- | --- | --- | --- | --- | --- | --- | --- | --- | --- |
| *SON* | 21:34923773 C>G | Heterozygous, exonic, nonsynonymous SNV | NM_001291411, exon3,  c.2236C>G, p.L746V | Unknown variant | 17.6 | Damaging | Neutral | Probably Damaging | Disease Causing | Deleterious | Damaging | Pathogenic | ZTTK syndrome |
| *EVC* | 4:5720981 G>A | heterozygous, exonic, splicing, nonsynonymous SNV | NM_001306090, exon2,  c.181G>A, p.D61N | Unknown variant | 24.4 | Damaging | Damaging | Neutral | Polymorphism | Neutral | 0.009 | Probable polymorphism | Weyers acrofacial dysostosis, Ellis-van Creveld syndrome; AR |

**†** Japanese and Finnish populations

AR, autosomal recessive

SNV, single nucleotide variant

MAF, minor allele frequency

hg19, reference genome
